# Prenatal diagnosis of persistent left superior vena cava: a retrospective study of associated congenital anomalies

**DOI:** 10.4274/tjod.galenos.2019.02679

**Published:** 2019-03-27

**Authors:** Mehmet Özsürmeli, Selim Büyükkurt, Mete Sucu, Erol Arslan, Çiğdem Akçabay, Selahattin Mısırlıoğlu, Masum Kayapınar, Nazan Özbarlas, Süleyman Cansun Demir, Cüneyt Evrüke

**Affiliations:** 1 University of Health Sciences, Derince Training and Research Hospital, Clinic of Obstetrics and Gynecology, Kocaeli, Turkey; 2 Çukurova University Faculty of Medicine, Department of Obstetrics and Gynecology, Perinatology Unit, Adana, Turkey; 3 Çukurova University Faculty of Medicine, Department of Pediatrics, Pediatric Cardiology Unit, Adana, Turkey

**Keywords:** Congenital anomaly, superior vena cava, prenatal diagnosis, ultrasound

## Abstract

**Objective:**

To evaluate persistent left superior vena cava (PLSVC) cases according to associated cardiac, extracardiac, and chromosomal anomalies in the prenatal period and to review their outcomes.

**Materials and Methods:**

The data of patients with a prenatal diagnosis of PLSVC between January 2013 and December 2017 were reviewed retrospectively.

**Results:**

Data of 32 cases were reviewed. Nineteen (60%) cases were associated with cardiac defects, 5 (15%) were associated with both cardiac and extracardiac defects, and 8 (25%) had no associated anomalies. Two fetuses had karyotype anomalies. All patients with isolated PLSVC survived. Among the cases associated with extracardiac anomalies, cardiac anomalies, and with both extracardiac and cardiac anomalies, the survival rate was 40%, 40%, and 25%, respectively. Outcome was more favorable in cases with isolated PLSVC (100% vs. 40%).

**Conclusion:**

Prenatally diagnosed PLSVC is associated with cardiac and extracardiac anomalies in the majority of cases. The prognosis is good in isolated cases, but worsens when accompanied by cardiac or extracardiac anomalies.

**PRECIS:** In the present study, we evaluated prenatally diagnosed cases of persistent left superior vena cava according to associated anomalies.

## Introduction

Persistent left superior vena cava (PLSVC) is the most common variant of the thoracic venous system^(1)^. It was observed as 0.3% in autopsy series and in 4-8% of patients with congenital heart diseases^(2)^. In the embryologic period, it is thought that a defect in left cardinal vein regression results in PLSVC^(3,4)^. PLSVC usually drains via the coronary sinus into the right atrium. In a few cases, it drains to the left atrium^(5)^. There is no significant hemodynamic effect of isolated PLSVC because systemic venous blood in the majority of cases drains to the right heart^(6,7,8)^. However, it is associated with cardiac and extra cardiac anomalies in 83% and 48% of cases, respectively^(3,8)^. A prenatal diagnosis of PLSVC is very important for proper management and counseling.

In this study, we aimed to identify prenatally diagnosed cases of PLSVC in our clinic, to evaluate the associated cardiac, extracardiac, and chromosomal anomalies, and to review their outcomes.

## Materials and Methods

This retrospective study was conducted at Çukurova University Hospital, an academic tertiary referral center, in the Pediatric Cardiology and Prenatal Ultrasound Unit. All women who were diagnosed as having fetal PLSVC from January 2013 to December 2017 were analyzed. Data were collected from the digital patient recording system.

All sonographic evaluations were performed by one of the ten experienced authors, using a Voluson E6 and Voluson 730 with a convex volumetric transducer probe (RAB 6-D 2-7 MHz and RAB2 5L) (GE, Zipf, Austria). The study population was composed of patients who presented to our center for routine second-trimester anatomic screening and those referred to us for suspected anomalies (low risk, unselected patients). There was no family history in the study group.

**Figure 1:**
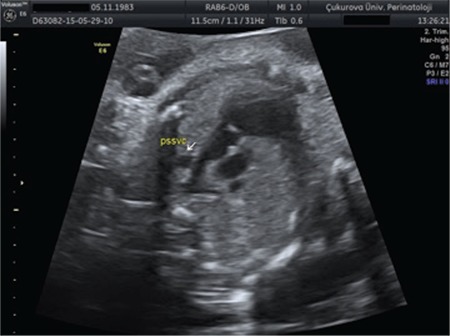
A persistent left superior vena cava in three-vessel view. It is seen as a fourth vessel to the left of the pulmonary artery

**Figure 2:**
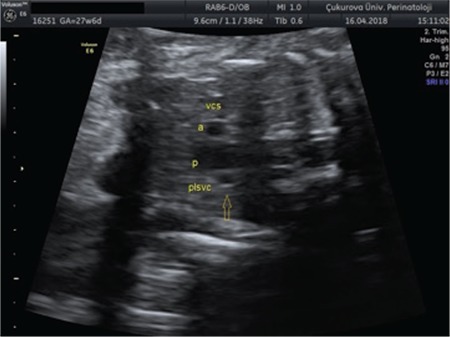
A persistent left superior vena cava in three-vessel trachea view

Fetal echocardiography was performed by visualizing standard anatomic planes. All cases were examined by a perinatologist and a pediatric cardiologist. The diagnosis of PLSVC was made with the presence of an extra vessel seen on the left side of the pulmonary artery in the three-vessels/three-vessels trachea view (Figure 1 and 2). The diagnosis was confirmed by showing the connection of the vessel to the coronary sinuses using gray scale and color Doppler in the long axis. The patients were compared in three groups (isolated cases, PLSVC associated with cardiac anomalies, and PLSVC associated with extracardiac anomalies). All parents were counseled based on the cardiac and extracardiac pathologic findings.

Hyperechogenic cardiac focus and dilated coronary sinuses that were thought to occur due to PLSVC were not classified into cardiac anomalies. In addition, soft markers such as pelviectasis, choroid plexus cyst, and nasal hypoplasia were not considered as fetal anomalies.

An invasive prenatal procedure for karyotype analysis was offered in the presence of malformations on ultrasound. In isolated PLSVC, an invasive procedure was not offered. For the cases without prenatal karyotyping, chromosome analysis was performed postnatally according to the clinical findings. If prenatal or postnatal chromosome analysis was not performed, the karyotype was considered to be normal in healthy infants. Prenatal diagnosis was confirmed through postnatal echocardiography or cardiac surgery in all surviving patients. The findings of all the cases with neonatal loss and termination of pregnancy (TOP) were confirmed by autopsy, except for cases in which the parents did not accept post mortem examination.

The outcome was considered favorable if the infant was alive and doing well, whereas TOP, in utero death, and neonatal death constituted unfavorable outcomes. All pregnant women were informed and written content was obtained. This study was approved by the Ethics Committee of the University of Çukurova.

Statistical analysis was performed using Fisher’s exact test and the Mann-Whitney U test. All values are given as mean ± SD. P<0.05 was considered to be significant.

## Results

Over the study period, a total of 33 fetuses with PLSVC were examined. One of patients was excluded due to incomplete data and loss to follow-up. Thirty-one out of the 32 patients had a normal right superior vena cava. Twenty-two cases were referred due to other cardiac and extracardiac defects, and PLSVC was detected during echocardiography. In ten cases, PLSVC was detected in our clinic during routine ultrasound scanning. The demographic characteristics, associated extracardiac findings, gestational weeks at diagnosis, karyotype and outcomes of the fetuses are shown in Table 1.

Group 1 (isolated cases) consisted of eight (25%) patients. Karyotype analysis was performed in two cases and all results were normal. The karyotype was considered to be normal in six healthy infants. All of the isolated cases are alive and well.

Group 2 (associated with cardiac anomalies) comprised 19 (60%) patients. Four out of the 19 patients also had extra cardiac anomalies. Karyotype analysis was performed in eight cases in the prenatal period; trisomy 21 was detected in two cases. The genetic status of the patients who do not accept karyotype analysis and autopsy (7 cases) is unknown.

Group 3 (associated with extracardiac anomalies) was constituted by five (15%) patients. Karyotype analysis was performed in two cases and all results were normal. The genetic status of one patient who died during the newborn period was unknown. The two fetuses with additional anomalies are alive and well. The rate of aneuploidy in the total cohort was 8.3% (2/24).

The outcome was significantly more favorable in group 1 compared with groups 2 and 3 (Table 1). Table 2 shows the outcome of the cases in detail. All patients with isolated PLSVC survived and were doing well at the time of writing. On the other hand, among the cases associated with extracardiac defects, cardiac defects, and both extracardiac and cardiac defects, the survival rate was 40%, 40%, and 25%, respectively. The oldest of the 17 surviving children is 5 years old. The mean follow-up period was 29 months. Nine patients underwent surgery for extracardiac defects or cardiac defects other than PLSVC, and five died postoperatively. The features of all cases in group 1, group 2 and group 3 are summarized in Tables 3, 4, and 5, respectively.

Five cases were associated with heterotaxy syndromes. After exclusion of patients with heterotaxy, most congenital heart defects were ventricular septal defects (six cases) and hypoplasic left heart (four cases).

## Discussion

In this study, we reviewed the prenatal and postnatal cardiac and extracardiac findings, associated chromosomal anomalies, and outcomes in 32 PLSVC cases.

PLSVC can be diagnosed easily during the perinatal period. In the three-vessel view, from the left to right side the pulmonary artery, aorta, and vena cava superior are seen with ultrasound. In addition to these three vessels in fetuses with PLSVC, a fourth vessel is seen on the left side of the pulmonary artery^(4)^. In addition, dilated coronary sinuses, slightly inferior of the four-chamber view, is known as an indirect sign of PLSVC^(9)^. It has to keep in mind that dilated coronary sinuses can also be seen in pulmonary venous anomalies, and so other views are needed for the confirmation of PLSVC^(9)^. The confirmation of the diagnosis is made by showing the draining of the vessel to the coronary sinuses on the left parasagittal view. Also, a dilated coronary sinus may be misdiagnosed as an ostium primum atrial septal defect at the level of the opening of the coronary sinus into the right atrium^(10)^. Differential diagnosis of PLSVC includes the supracardiac type of abnormal pulmonary venous connection. Color Doppler can help differentiate PLSVC from the vertical vein in supracardiac pulmonary venous connection. Blood flow is toward the heart in PLSVC and toward the opposite direction in the vertical vein^(11)^.

The most significant clinical implication of prenatally diagnosed PLSVC is the association with cardiac and extracardiac defects. It was reported that PLSVC was accompanied by additional cardiac anomalies in 80% of cases^(3,8)^. Moreover, in only 5-9% of cases, PLSVC was seen as an isolated finding. Isolated PLSVC was diagnosed more frequently in newer series^(12,13)^. In our series, 41% (13/32) of the cases were not associated with additional cardiac malformation and the rate of isolated PLSVC was found as 25% (8/32), which was consistent with the recent studies. The inconsistency of recent studies with previous studies may be a result of more common incorporation of the three-vessel view in routine systematic ultrasound examinations of fetal hearts in the last few years, as well the increasing awareness about diagnosing PLSVC.

In our series, septal defects were the most common cardiac defect, followed by hypoplasic left heart syndrome. Heterotaxy was observed in only 5 of the 19 cases with cardiac defects, being much lower than the other previous prenatal series in which it was reported as 41-54%^(3,8)^. The most frequent anomalies were reported to be ventricular septal defect and conotruncal anomalies in the study by Choi et al.^(13)^. However, they did not diagnose any heterotaxy syndrome in that study. Diagnosing PLSVC with minor anomalies in newer studies may support the idea of increased awareness of PLSVC.

It was thought that expansion of the lung and cardiac atriums was required for obliteration of the anterior cardinal vein^(14)^. However, the large variety of other associated cardiac malformations does not support this concept.

The rate of aneuploidy was 8% in our study, similar to previous series^(3,12)^. PLSVC is seen much more frequently in chromosomally abnormal fetuses than in normal fetuses. Du et al.^(15)^ reported that the odds ratio for PLSVC in chromosomally abnormal fetuses compared with normal fetuses was 27.5 (95% CI: 15.8-47.8)^(15)^. Nevertheless, it has to be considered that either cardiac or extracardiac anomalies are seen more frequently in chromosomally abnormal fetuses. Although isolated PLSVC is thought to be a benign vascular variant and has good prognosis, some authors have asserted that fetal karyotyping should be routinely offered whenever PLSVC is detected prenatally, based on the possibility of missing some cardiac anomalies and obscure extracardiac anomalies suggesting aneuploidy^(16,17)^. Although it was reported that the rate of abnormal karyotype was 7% in isolated cases, it would be difficult to recommend routine prenatal invasive procedure when PLSVC is isolated^(18)^. The first trimester combined risk for trisomies in isolated cases is not reported in many studies, and it might be possible that undetected anomalies are included in analyses^(18)^. Larger studies are needed in low-risk populations to establish whether isolated PLSVC should be an indication for fetal karyotyping^(13)^.

In our series, there were two trisomy 21 cases. Both cases had prenatally diagnosed cardiac anomalies. Trisomy 21 has been diagnosed either in isolated cases^(10,13)^, against it we could not find any trisomy 21 in isolated cases. The number of cases reported in the literature is insufficient to draw the conclusion that PLSVC should be accepted as a marker for trisomy 21.

The outcome of PLSVC is associated with other cardiac and extracardiac findings. In previous studies, all isolated cases had a favorable prognosis. Similarly, in our cohort, all fetuses with isolated PLSVC did well after birth, whereas the survival rate declined to 40% when an additional cardiac anomaly was observed, and to 25% when an extracardiac finding was also present. These findings support the suggestion that isolated PLSVC is a benign anomaly; however, there is a need to screen for cardiac and extracardiac anomalies.

The absence of the right superior vena cava may cause difficulties during a potential invasive procedure to vessels (e.g. pulmonary artery catheterization, systemic venous cannulation) in the adulthood. Therefore, it is important to diagnose the absence of the right superior vena cava. There was only one case of absent vena cava superior in our study.

The limitations of our study are its retrospective character, the underdiagnosis of isolated PLSVC in referred cases, and the higher incidence of complicated cases due to the reference nature of our center. In a fetus with multiple anomalies, attention is given to the anomalies and therefore PLSVC may not be diagnosed and this can cause a lower incidence than the real incidence. We do not know the real rate of false-negative cases because postnatal echocardiography was not routinely performed on neonates without a prenatal diagnosis of coronary heart disease. Furthermore, the karyotype status of fetuses for whom the parents did not accept autopsy were unknown. However, despite these limitations, with this study we underline the significance of the three-vessel view during routine fetal screening and draw attention to the increasing incidence of isolated PLSVC in clinical practice.

In conclusion the prognosis is good in isolated cases, while getting worse when accompanied to cardiac or extracardiac anomalies. The prenatal diagnosis of PLSVC is important and once it is diagnosed a detailed anatomic screening and investigation of chromosomal anomalies have to be done.

## Figures and Tables

**Table 1:**
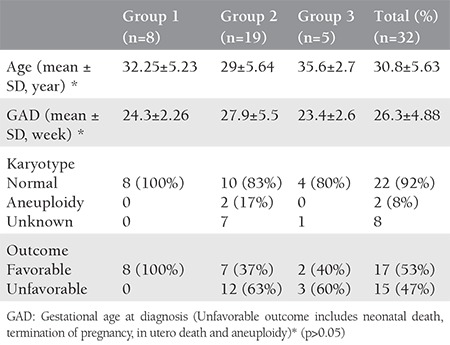
Demographic and clinical features of the cases

**Table 2:**
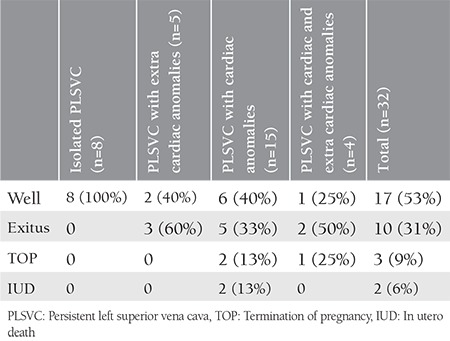
Outcome of the cases

**Table 3:**
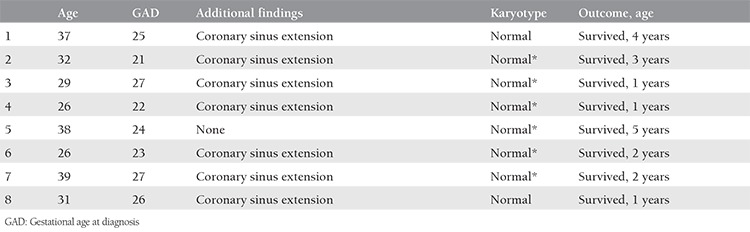
Features of isolated persistent left superior vena cava cases

**Table 4:**
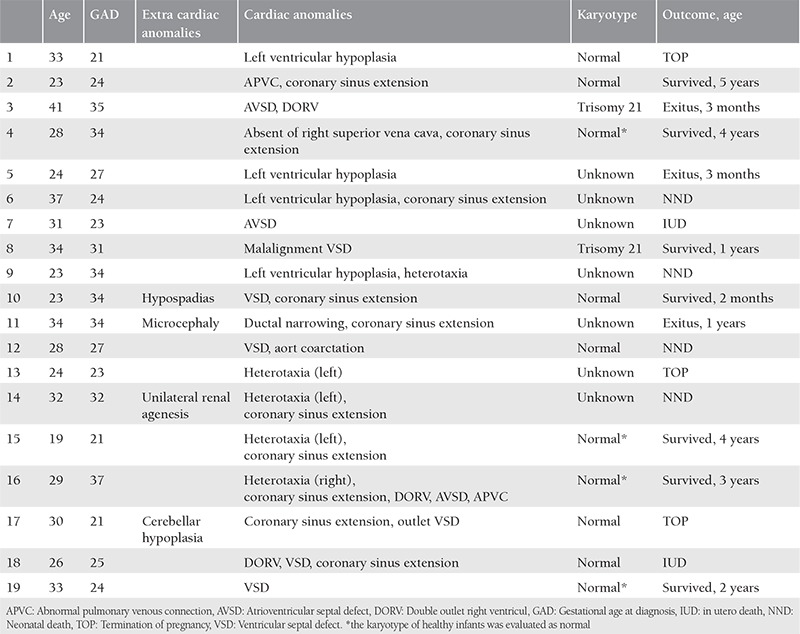
Features of cases associated with cardiac anomalies

**Table 5:**
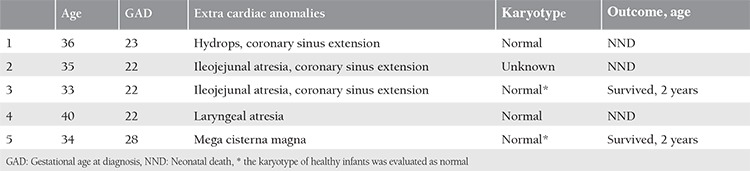
Features of cases associated with extra cardiac anomalies
